# Thrombotic microangiopathy multidisciplinary assessment team: demographics, final diagnosis, treatment, and outcomes

**DOI:** 10.1186/s12882-025-04446-z

**Published:** 2025-09-26

**Authors:** Matthew Nguyen, Gayathri Dileep, Samir Patel, Fawaz Al Ammary, Minh-Ha Tran, Benjamin J. Lee, Stefan Ciurea, Sheetal Desai, Alpesh Amin, Hongyu Zhao, Omid Vadpey, Dao Le, Tai Truong, Antoinette Abdelmalek, Jordan Perkins, Rebecca Ahdoot, Fatima Malik, Antoney Ferrey, Uttam Reddy, Ekamol Tantisattamo, Yongen Chang, Wei Ling Lau, Yulian Khagi, Lisa Lee, Zahra Pakbaz, Ramy M. Hanna

**Affiliations:** 1 Department of Medicine, Division of Nephrology, Hypertension and Kidney Transplantation, University of California Irvine, Irvine, CA USA; 2 Department of Medicine, Division of Transfusion Medicine, University of California Irvine, Irvine, CA USA; 3 Department of Medicine, Division of Hematology Oncology, University of California Irvine, Irvine, CA USA; 4 Department of Medicine, Division of Rheumatology, University of California Irvine, Irvine, CA USA; 5 Department of Medicine, University of California Irvine, Irvine, CA USA; 6 Department of Medicine, Division of Nephrology, University of California Irvine - VA LBMC, Irvine, CA USA; 7 Department of Medicine, City of Hope, Duarte, CA USA

**Keywords:** Thrombotic Microangiopathy (TMA), Multidisciplinary team, Complement-Mediated Thrombotic Microangiopathy (CM-TMA), Rare disorder, Thrombotic thrombocytopenic purpura, Hematopoietic stem cell transplant, Nephrology, Hematology, Glomerulonephritis

## Abstract

**Rationale & objective:**

Thrombotic Microangiopathies (TMAs) include an etiological diverse group of phenotypically similar disorders. While individually rare, they are seen as an aggregate with regularity. Prior reports suggested the importance of approaching TMAs in a multidisciplinary fashion. We present the development and data over a 4-year period after establishing the University of California Irvine (UCI) TMA team.

**Design, settings, participants, and measurements:**

This is a single-center retrospective case observational study of 101 diverse patients demonstrating demographics, diagnoses, triggers, treatments applied, and outcomes representing a wide range of hematologic pathologies.

**Findings:**

Of the 101 patients, 47 were females with an age range between 18 and 83 years old. 46 of our 101 patients were diagnosed with TMA. 24 patients were diagnosed with atypical hemolytic uremic syndrome (aHUS). Eight patients were diagnosed with Hematopoietic Stem Cell Transplant TMA (HSCT-TMA). Of the 24 aHUS patients, 16 were treated and 8 were evaluated for complement blockade. Of those treated, 100% demonstrated a hematologic response, 81.3% had initial renal remission and 68.8% of those who were on dialysis (11 of 16) remain off dialysis. Of the 8 HSCT-TMA patients, 5 were treated and 3 were evaluated for complement blockade. Of those treated, 80% demonstrated a hematologic response, 100% had initial renal remission; however, of all treated patients eventually returned to needing dialysis.

**Limitations and conclusions:**

This observational study highlights the importance of the TMA multidisciplinary team to improve diagnosis and optimize outcomes of this rare condition. Future research is needed to subdivide response to various TMA to current and emerging therapies in the context of multidisciplinary care.

**Clinical trial number:**

Not applicable.

**Supplementary Information:**

The online version contains supplementary material available at 10.1186/s12882-025-04446-z.

## Introduction

Thrombotic Microangiopathies (TMAs) are a group of etiologically disparate diseases that appear similar clinically. However, they may involve various degrees of thrombocytopenia, microangiopathic hemolytic anemia (MAHA), and acute kidney injury (AKI) of varying severity [1]. These may occur in the context of a genetic predisposition, a mix of genetic and environmental factors, or due to purely environmental triggers [1–3]. Triggers are extremely varied and underlying them often are disorders in coagulation proteins, genetic mutations in complement proteins, auto antibodies affecting various coagulation/complement cascades, overwhelming adaptive immunity activation, inborn errors of metabolism amongst other complex pathologies [4].

A complex group of diseases such as these require multiple disciplines and a team effort from equally diverse specialists [5]. The needed expertise may include hematologists, transplant nephrologists, transplant hematologists, nephrologists, pharmacists, administrators, health care executives, rheumatologists, maternal-fetal medicine (obstetrical) specialists, and infectious disease experts [1, 5]. Within our institution at the University of California Irvine (UCI), the TMA team consisted of nephrology, benign hematology, transplant hematology, transfusion medicine, internal medicine, rheumatology, and a pharmacy liaison [1]. Due to the high cost of the therapy, processes were in place to assist with the approval, authorization, and acquisition of the medication. This team was built from an already active publishing faculty from UCI with an ongoing interest in TMA diagnosis and treatment [1–4, 6–12]. A group of interested medical students, residents, interested fellows were also utilized to collate research results for dissemination and publication, led by the lead author and overseen by the senior author.

The TMA team is activated by consultation upon a positive hemolysis screen sent by emergency staff, hospitalists, internists, other specialists in the setting of renal failure and thrombocytopenia. The TMA team operates on a stand by basis, but whenever a particularly challenging case comes up, we do convene both via zoom and in person on a scheduled basis, to form a ‘TMA’ board. When institutional support becomes available the plan is for a standing TMA board meeting monthly is planned.

We present our data from the last 4 years as an active TMA team and discuss how our team functions. The aim of the work at our institution and this report is fourfold:

I) To describe the function and purpose of a multidisciplinary TMA team
II) To present and list our criteria for the definition and diagnosis of patients with TMA along with our diagnostic algorithm
III) To present the varying types of cases that were referred to our team with concerns of TMA and the overall underlying diagnosis
IV) To present the data on the multiple classes of agents that were used and the outcomes upon discontinuation of complement blockade

## Methods

**Fig. 1:**
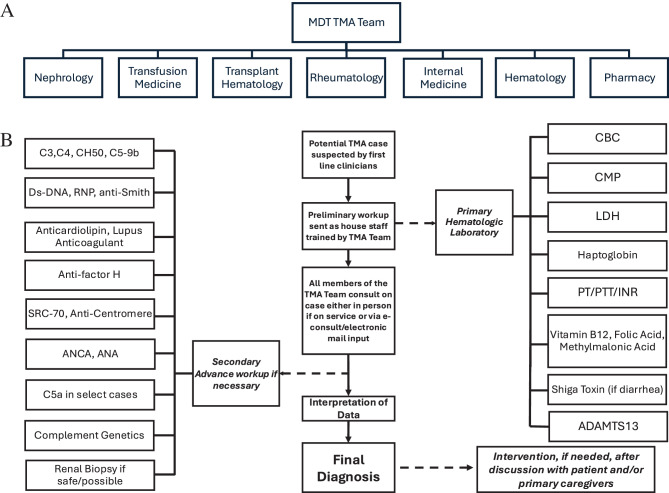
MDT TMA team organization. (**a**) MD, medical doctor; PharmD, doctor of pharmacy, TMA, thrombotic microangiopathy. MDT, multidisciplinary team. TMA team activation and TMA workup. (**b**) ADAMTS13, a disintegrin and metalloproteinase thrombospondin type 1 motif 13; ANCA, anti-neutrophil cytoplasmic antibody; ANA, anti-nuclear antibody; C3, complement factor 3; C4 complement factor 4; C5a, anti C5a (split product of C5); C5-9b, membrane attack complex level; CBC, complete blood count; CH50, total complement hemolytic activity; CMP, comprehensive metabolic panel; DsDNA, anti-double stranded deoxyribonucleic acid; INR, international normalized ratio; LDH, lactate dehydrogenase; PT, prothrombin time; PTT, activated partial thromboplastin time; RNP, anti-ribonucleic protein; SRC 70, anti-topoisomerase; TMA, thrombotic microangiopathy

This study used retrospective data on 101 adult patients identified from consult logs while inpatient for suspected TMA with matching of at least 4 of the following criteria of hemolytic anemia (Hb < 10 g/dL), thrombocytopenia (platelet count < 150,000/uL), elevated lactate dehydrogenase (LDH > 280 U/L), decreased haptoglobin ( < 30 mg/dL),and signs of acute organ failure, prompting a consult to one or more of the TMA team members at University of California, Irvine from 01/2020 to 07/2024. Of note mild to many schistocytes were helpful in ruling in the presence of hemolytic anemia; however, was not very specific to the diagnosis of TMA as its levels can wax and wane in the acute phase. The multidisciplinary team included providers across the following specialties: nephrology, transfusion medicine, hematology, rheumatology, internal medicine, and pharmacy as shown in Fig. 1. Patient cases were reviewed in person in the inpatient setting, or if off site, through secure emails, calls and integrated electronic medical records messaging service. Patients identified with signs of TMA received standardized lab algorithm as shown in Fig. 1.

The suggested workup was divided into two steps, the preliminary workup activated upon recognition of a TMA, and a confirmatory workup to evaluate triggers, examine complement genetics (for prognosis), as well as in some cases evaluating C5-9b, C5a, drug levels, and complement functional assays when warranted [1–5] (Fig. 1). Education on both arms of this workup were provided by the TMA team members extensively to colleagues, the house staff, the community allowing multiple tertiary care referrals to be sent to us as an emerging TMA center [1, 3, 4]. Following secondary/confirmatory work-up a multidisciplinary team meeting was held for further evaluation of diagnostic workup and possible intervention. While UCI has had some TMA expertise since 2020 and prior, the UCI TMA team was more formally established and institutionally recognized in 2021, resulting in firmer protocols in diagnosing patients with concerns of TMA and greater growth. This development has also attracted opportunities in teaching, translational research, and also increased clinical trials opportunities [1] (Supplemental Fig. 1).

These patients had a large database of information collected including, age, gender, TMA team diagnosis, therapy, hematological outcome, renal replacement therapy need, renal outcome, survivorship, as well as various other biomarkers such as auto antibodies, hemoglobin, platelets, lactate dehydrogenase, coagulation profiles, a disintegrin and metalloproteinase thrombospondin motif 13 member 1 (ADAMTS13) levels that are to be described in subsequent articles. These data were all useful in the usual clinical care of patients whereby they were clinically identified as being more likely to have on TMA versus another etiology (i.e. Coppo et.al. data on TTP vs. aHUS); or in calculating PLASMIC scores for plasmapheresis [13, 14]. Genetic testing when warranted was done by Machaon Diagnostic Laboratory.

The current form that genetic testing utilizes are send out testing to the Iowa Complement Lab and Machaon Lab. While the Machaon lab relies on Polymerase Chain Reaction (PCR) analysis for known disease causing alleles, Iowa typically utilizes the more accurate but more time-consuming whole exome sequencing. PCR allele mutation analysis, while not 100% perfect, is faster. The rapid speed of return of the PCR analysis in 2–3 weeks is deemed helpful to our team.

For all treated patients with active disease (which is noted as presence of hemolytic anemia, thrombocytopenia, end organ disease, including extra-renal manifestation), hematological and renal response were recorded for patients with complement mediated TMA (CM-TMA) who received complement blockade therapy. Hematological response is defined as normalization of hemoglobin and platelet values and cessation of signs of hemolytic anemia. Renal response is denoted as normalization or stabilization of labs representing kidney function and is also recorded for patients upon cessation of dialysis. For peri-transplant blockade for patients who are on dialysis, the protocol in our center is to initiate complement blockade several weeks before transplant. Patients with extra-renal symptoms, particularly ongoing thrombophilia, are also to be considered for complement blockade with possible addition of anti-coagulation. Thresholds for not using therapy include patients with end-stage renal disease without extra-renal manifestations; however, these patients will receive treatment with peri-transplant cooperation with our transplant team whether at UCI or other transplant centers.

In general, our protocol in all treated patients is a minimum of 2 weeks of penicillin VK, cephalosporin, or macrolide (azithromycin) therapy as prophylaxis when initiating complement inhibitors [1–4]. At that time vaccination with Pneumococcus vaccine, *Haemophilus Influenza* vaccine, Meningococcus serotype A and B vaccinations with appropriate booster doses are recommended [15, 16]. This is the vaccination regimen being given with antibiotics as Immunoglobulin (IgG) formation takes 2 weeks from initial Immunoglobulin M (IgM) formation. Antibiotics could be continued longer than 2 weeks in patients who are high risk and in some unvaccinated/boosted patients could be extended to include length of treatment plus 90 days^15,16^. Regarding our treatment protocol for our aHUS and HSCT-TMA patients, we utilize the 4 following agents: Eculizumab, Ravulizumab, Narsoplimab and Iptacopan. For Eculizumab, the dosing schedule begins with 900 mg IV once a week for the first four weeks followed by a fifth dose of 1200 mg IV one week after. For maintenance the regimen consists of 1200 mg every 2 weeks. For Ravulizumab, dosing is weight based dependent and is a loading dose based on the following weights: patients weighing 40 to < 60 kg receives 2400 mg IV, those ≥ 60 to < 100 kg receives 2700 mg IV, and patients ≥100 kg receive 3000 mg IV. For maintenance dosing, we initiated 2 weeks after the loading dose and is also dependent on the patients’ weight: 3000 mg IV every 8 weeks for patients 40 to < 60 kg, 3300 mg IV every 8 weeks for those ≥ 60 to < 100 kg, and 3600 mg IV every 8 weeks for patients ≥100 kg. For Narsoplimab dosing varied dependent on patient, one of which was 370 mg every 72 hours while the other was 370 mg twice weekly. In both cases, laboratory markers were assessed frequently for response. Finally, Iptacopan is administered orally at a dose of 200 mg twice daily.

The rationale behind non c5 blocking agent use is as follows: a mannan binding lectin serine protease inhibitor − 2 (MASP 2 inhibition) is being developed specifically for HSCT-TMA (by Omeros Pharmaceuticals) and is in preparation for further evaluation by the US Food and Drug Administration (US-FDA) and our Bone Marrow Transplant (BMT) unit has been an early adopter and partner of theirs in testing via the Compassionate Use Authorization (CUA) FDA managed access program.

The rationale for Factor B inhibition is the active trials testing iptacopan (Factor B blocking agent) Novartis’ agent that is in active clinical trials for aHUS. Factor B blockade has since been approved for C3GN, paroxysmal nocturnal hemoglobinuria (PNH), and is being evaluated in trials for Immunoglobulin A Nephropathy (IgAN) and Immune complex-membranoproliferative glomerulonephritis (IC-MPGN).

### Statistical analysis

The data was analyzed for demographics, diagnosis, treatment, and outcome and is presented to provide greater insight into the experience our growing TMA team encountered as we matured into a cohesive force. We calculated percentages and other very basic statistical mathematical analysis including averages, standard deviations, medians, and student t tests in excel.

This study was approved by Institutional Review Board (IRB) [UCI IRB protocol #2975] covering data from 01/2020–07/2024.

## Results

The collected data indicated 101 patients were evaluated at UCI between 2020 and 2024. The 18–30-year-old group had 22 (21.8%), the 31–43 years of age group had 18 (17.8%), the 44–56 years of age group had 17 (16.8%), the 57–69 years of age group had 25 (24.8%), the 69+ years of age group had 19 (18.8%). There were 47 females (46.5%) and 54 males (53.5%) (Table 1).

**Table 1:** Gross demographic data

Gross demographic	Total (*n* = 101)
Age (years)	
18–30	22 (21.8%)
31–43	18 (17.8%)
44–56	17 (16.8%)
57–69	25 (24.8%)
69+	19 (18.8%)
Gender	
Male	54 (53.5%)
Female	47 (46.5%)
Diagnosis	
TMA	46 (45.6%)
ATN	7 (6.93%)
Anemia of CKD	6 (5.94%)
Preeclampsia	4 (3.96%)
Sepsis	3 (2.97%)
ITP	3 (2.97%)
Blood Loss	3 (2.97%)
Malignant Hypertension	3 (2.97%)
Drug Induced Pancytopenia	2 (1.98%)
DIC	2 (1.98%)
Cirrhosis	2 (1.98%)
APLS/SLE	2 (1.98%)
AML	2 (1.98%)
Type III Hypersensitivity	1 (1.00%)
TLS	1 (1.00%)
Shock	1 (1.00%)
Scleroderma Renal Crisis	1 (1.00%)
PANDAS	1 (1.00%)
Obstruction and TKI	1 (1.00%)
MGUS	1 (1.00%)
Immune Complex Glomerulonephritis	1 (1.00%)
Hemolytic Anemia due to Impella	1 (1.00%)
Hemolysis due to G6PD Deficiency	1 (1.00%)
ECMO Induced Hemolysis	1 (1.00%)
Congestive Hepatopathy	1 (1.00%)
Shock (Cardiogenic)	1 (1.00%)
C3 MIDD	1 (1.00%)
C3GN + Factor H aB	1 (1.00%)
AAV Gene Therapy suspected TMA	1 (1.00%)
APLS Positive	9 (9.38%)
Confirmed Pathogenic Mutation (Non-VUS out of 46)	7 (15.2% out of 46 total)

This table documents the overall demographics of all the patients referred to the team concerning TMA. It summarizes the final diagnosis in each of the referrals, their age range, and their gender. This table also documents the percentage of patients with positive APLS and confirmed pathogenic mutation

Ab = antibody; aHUS = atypical hemolytic anemia; TMA = thrombotic microangiopathy; ATN = acute tubular necrosis, APLS = Antiphospholipid syndrome; SLE = systemic lupus erythematosus; HTN = hypertension, CKD = chronic kidney disease; AML = acute myelogenous leukemia; DIC = disseminated intravascular coagulation; ITP = immune thrombocytopenic purpura; MGUS = monoclonal gammopathy of undetermined significance; C3GN = C3 glomerulonephritis; MIDD = Monoclonal immunoglobulin deposition disease; ECMO = extracorporeal membrane oxygenation; G6PD = Glucose 6 phosphate dehydrogenase; TKI = Tyrosine Kinase Inhibitor; PANDAS = Pediatric Autoimmune Neuropsychiatric Disorders Associated with Streptococcal Infections; TLS = Tumor Lysis Syndrome; VUS = Variant of Undetermined Significance

In our cohort of 101 patients, 46 (45.6%) of our patients were diagnosed with TMA. Acute tubular necrosis (ATN) was diagnosed in 7 cases (6.93%), anemia of chronic kidney disease (CKD) was diagnosed in 6 (5.94%) cases, and preeclampsia was diagnosed in 4 (3.96%) of cases. Sepsis was diagnosed in 3 (2.97%) cases, Immune thrombocytopenia purpura (ITP) was diagnosed in 3 (2.97%) of cases, and massive blood loss was diagnosed in 3 (2.97%) of cases. Malignant hypertension (MHT) was diagnosed in 3 (2.97%) of cases), thrombotic thrombocytopenia purpura (TTP) was diagnosed in 2 cases (1.98%) of cases (counted under TMA), and drug induced pancytopenia was diagnosed in 2 (1.98%) of cases. Disseminated intravascular coagulation was diagnosed in 2 (1.98%) cases, cirrhosis was diagnosed in 2 (1.98%) of cases, and antiphospholipid syndrome (APLS)/systemic lupus nephritis (SLE) was diagnosed in 2 cases (1.98%). Acute myelogenous leukemia (AML) was diagnosed in 2 cases (1.98%), type 3 hypersensitivity reaction was diagnosed in 1 (1.00%) case, and tumor lysis syndrome was diagnosed in 1 (1.00%) case. Nonspecific shock was diagnosed in 1 case (1.00%), scleroderma renal crisis (SRC) was diagnosed in 1 (1.00%) case, and pediatric autoimmune neuropsychiatric disorders associated with streptococcal infection (PANDAS) were diagnosed in 1 case. Post obstructive AKI vs possible tyrosine kinase inhibitor toxicity was diagnosed in 1 case (1.00%), monoclonal gammopathy of undetermined significance was diagnosed in 1 case (1.00%), and immune complex glomerulonephritis was diagnosed in 1 (1.00%) case. Impella induced hemolytic anemia was diagnosed in 1 (1.00%) case, hemolysis due to glucose 6 phosphate deficiency (G6PD) was diagnosed in 1 (1.00%) case, and extracorporeal membrane oxygenation hemolysis was diagnosed in 1 (1.00%) case. Congestive hepatopathy was diagnosed in 1 (1.00%) case, cardiogenic shock was diagnosed in 1 (1.00%) case, and C3 glomerulonephritis with monoclonal immunoglobulin deposition disease (C3 MIDD) was diagnosed in 1 (1.00%) case. C3 glomerulonephritis with concurrent Factor H antibody was seen in 1 case (1.00%) and adeno-associated virus (AAV) gene therapy suspected TMA was diagnosed in 1 (1.00%) case. A total of 9 (9.38%) of patients were confirmed to be APLS Positive and 7 (7.29%) of patients had a confirmed pathogenic in our entire cohort of 101 patients (Table 1).

Genetic analysis was sent on the 46 patients with suspected treatable TMA types, of those 46, 7 had positive mutations for alternative complement genes (15.2%). The most common (3) were Complement factor H related protein 1, and CHFR 1–3 deletions (CFH1, CFHR1–3), CF3 (factor 3), Membrane cofactor protein (MCP), (Factor H) were all included in our cohort. We counted clear mutations showing pathogenic coding, and held off counting variants of uncertain significance (VUS) for now. This is probably why the rate of mutations is lower than the usually reported rate. In the future, we concede that these VUSs maybe proven conclusively to cause disease and we await such confirmation.

**Fig. 2:**
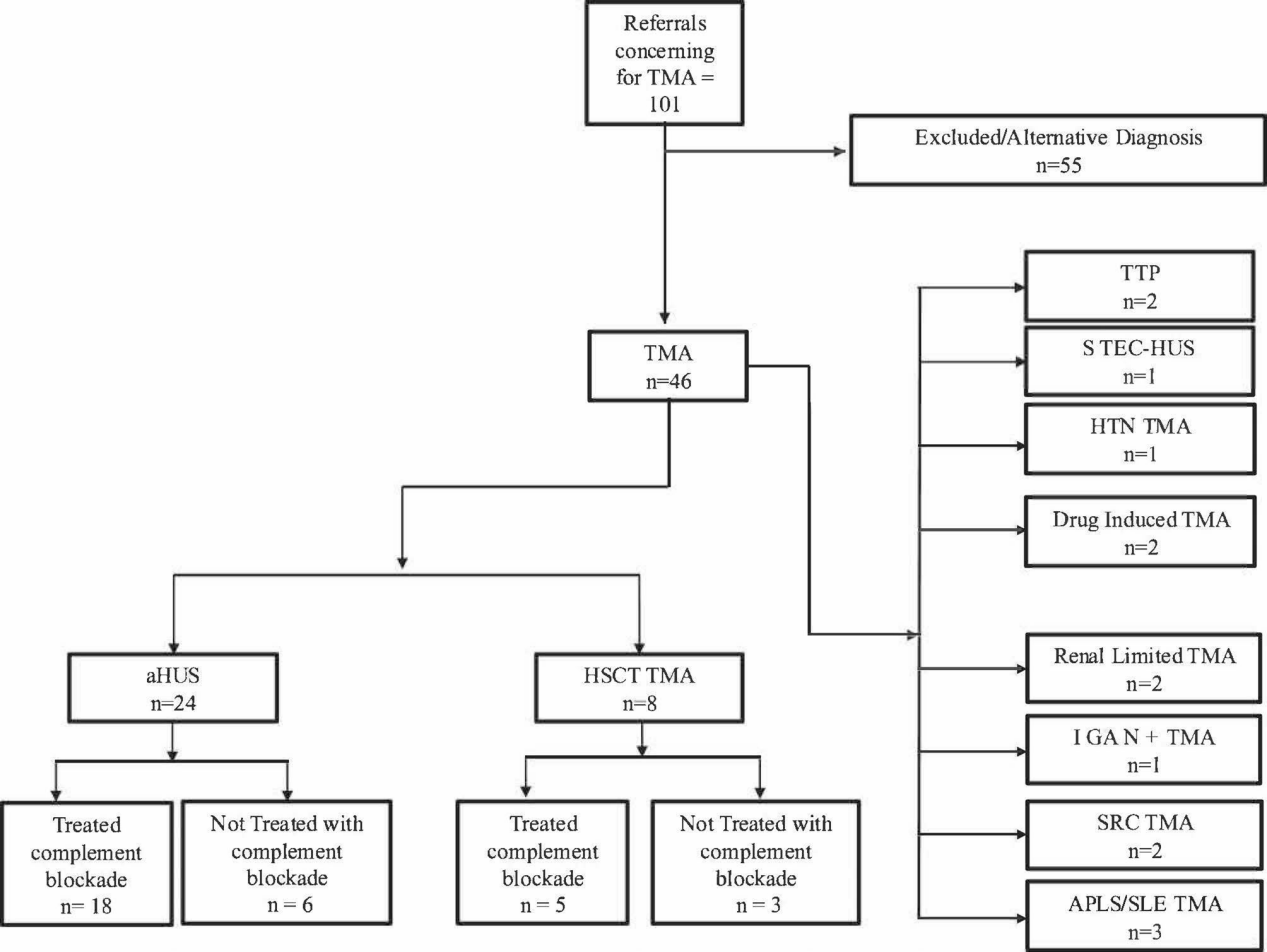
Referrals concerning for TMA, final diagnosis, and treatment. TMA, thrombotic microangiopathy; ETEC-TMA, enterotoxigenic; HTN, hypertension;IGA, immunoglobulin A; SRC, scleroderma renal crisis; APLS, antiphospholipid syndrome, SLE = systemic lupus erythematosus; HSCT, hematopoietic stemcell transplant; aHUS = atypical hemolytic uremic syndrome

In our cohort of 101 patients referred to our team concerning for TMA, there were 46 patients after workup confirmed to have TMA. In this TMA group, the 18–30-year-old group had 18 (39.1%), the 31–43 years of age group had 8 (17.4%), the 44–56 years of age group had 7 (15.2%), the 57–69 years of age group had 9 (19.6%), the 69+ years of age group had 4 (8.70%). There were 26 females (56.5%) and 20 males (43.5%). (Table 2) and (Fig. 2).

**Table 2:** TMA diagnosis and demographic

Gross demographic	Total (*n* = 46)
Age (years)	
18–30	18 (39.1%)
31–43	8 (17.4%)
44–56	7 (15.2%)
57–69	9 (19.6%)
69+	4 (8.70%)
Gender	
Male	26 (56.5%)
Female	20 (43.5%)
Subset of TMA	
aHUS	24 (52.2%)
HSCT TMA	8 (17.4%)
APLS/SLE TMA	3 (6.52%)
SRC TMA	2 (4.35%)
Renal Limited TMA	2 (4.35%)
Drug Induced TMA	2 (4.35%)
TTP	2 (4.35%)
HTN TMA	1 (2.17%)
-STEC-HUS	1 (2.17%)
IGAN+ TMA	1 (2.17%)
Lab Values	
Creatinine (mg/dL) (mean, std)/[median]	3.12, ±2.39 [2.35]
Platelet (thous/mcl) (mean, std)/[median]	124.66, ±108.48 [85]
Schistocytes (n, %)	5 (10.87%)
Hemoglobin (g/dL) (mean, std)/[median]	8.69, ±2.30 [8.25]
LDH (U/L) (mean, std)/[median]	715.74, ±761.43 [461]
Haptoglobin (mg/dL) (mean, std)/[median]	83.21, ±133.69 [ < 30]
Protein/Creatinine Ratio (mg/g) (mean/std)/[median]	3842.88, ±4133.27 [2191]
ICU Status (n, %)	26 (56.52%)
Need for CRRT (n, %)	8 (17.39%)
Newly Diagnosed aHUS/TMA patients per year emergently initiated on complement blockade prior to 2020 vs after 2020 and MDT-team implementation [mean number of patients ± std, (p-value]	1.6, ±0.5 vs 4.2 ± 1.9 (*p* = 0.020) [2 vs 7]

This table documents the overall demographics of our TMA population including their age range, their reported gender, and the subset diagnosis of these TMA patients. Furthermore, this table documents the average lab values and standard deviations of key labs reflecting renal function and signs of TMA. This table also documents the number of patients newly diagnosed with aHUS emergently initiated on Eculizumab prior to 2020 and after 2020. [median values], for minimum and maximum values see text

aHUS = atypical Hemolytic Uremic Syndrome; TMA = Thrombotic Microangiopathy; ATN = Acute Tubular Necrosis, HSCT = Hematopoietic Stem Cell Transplantation-associated; APLS = Antiphospholipid; SLE = Systemic Lupus Erythematosus; HTN = Hypertension; SRC = Scleroderma Renal Crisis; TTP = Thrombotic Thrombocytopenic Purpura; STEC-HUS=Shigatoxin producing ETEC = Enterotoxigenic Escherichia coli; IGAN = Immunoglobulin A Nephropathy

In our group of 46 TMA patients, 24 (52.2%) were determined to have atypical hemolytic uremic syndrome (aHUS), 8 (17.4%) were diagnosed with hematopoietic allogenic stem cell transplant induced TMA, 3 (6.52%) were diagnosed with antiphospholipid (APLS)/systemic lupus nephritis (SLE) TMA, and 1 (2.17%) patients were diagnosed with hypertensive (HTN) TMA. 2 (4.35%) patients were diagnosed with scleroderma renal crisis (SRC) induced TMA, 2 (4.35%) patients were diagnosed with renal-limited TMA, and 2 (4.35%) patients were diagnosed with drug induced TMA. 2 (4.35%) patients were diagnosed with thrombotic thrombocytopenic purpura (TTP), 1 patient (2.17%) was diagnosed with TMA from Shiga Toxin-Producing Escherichia coli-Hemolytic Uremic Syndrome (STEC-HUS) and 1 (2.17%) patient was diagnosed with immunoglobulin A Nephropathy (IGAN) with TMA.

Average creatinine of our TMA cohort was 3.12 mg/dl (±2.39) [median 2.35 mg/dL, minimum-maximum: 0.3–11.1 mg/dL], average platelet was 124.66 thousand/mcl (±108.48) [median 89 thousand/microliter (mcl), minimum-maximum 8–385 thousand/mcl], and average hemoglobin was 8.69 g/dL (±2.30) [median 8.25 g/dL, minimum-maximum 3.3–14.9 g/dL]. Average lactate dehydrogenase (LDH) in our TMA cohort was 715.74 U/L (±761.43) [median 461 U/L, minimum to maximum 106–3908 U/L], average haptoglobin levels was 83.21 mg/dL (±133.69) [median < 30 mg/dL, minimum to maximum < 30 (0) to 600 mg/dL], and average protein/creatinine ratio was 3842.88 mg/g (±4133.27) [median 2191 mg/g minimum to maximum 191 to 15,394 mg]. 5/46 patients were considered to have notable amount of schistocyte (greater than mild). 26/46 patients were admitted to ICU. 8/46 of our TMA patient cohort required continuous renal replacement therapy (CRRT).

The average number of newly diagnosed aHUS/TMA patients emergently initiated on complement blockade (usually eculizumab) per year in the hospital setting prior to 2020 was 1.6 (±0.5) [median 2015–2020 2, minimum-maximum 1–2] and the average number of newly diagnosed aHUS patients emergently initiated on eculizumab after 2020, and post-team implementation was 4.2 (±1.9) [median 2021–2025 5, minimum-maximum 2–7] with a statistically significant p-value comparison of 0.020.

The triggers identified for our cohort of 24 patients included 7 (29.2%) with systemic lupus erythematosus (SLE), 1 (4.17%) with APLS (antiphospholipid antibody syndrome), 5 (20.8%) with idiopathic/genetic aHUS/CM-TMA (no trigger identified), 4 (16.7%) had hypertension induced aHUS that did not resolve with hypertension treatment, 3 (12.5%) had post-partum TMA, 2 (8.33%) had aHUS associated with COVID-19, 1 (4.17%) had Covid-19 associated with an intraabdominal infection (cholecystitis), and 1 (4.17%) had aHUS associated with inflammatory bowel disease (IBD) (Crohn’s colitis).

Table 3 records that 100% of the patients diagnosed with CM-TMA/aHUS who needed to be treated with complement inhibitors had experienced AKI and 15 (93.8%) needed renal replacement therapy (RRT) at some point in their clinical course. (See Table 3, Fig. 2).

**Fig. 3:**
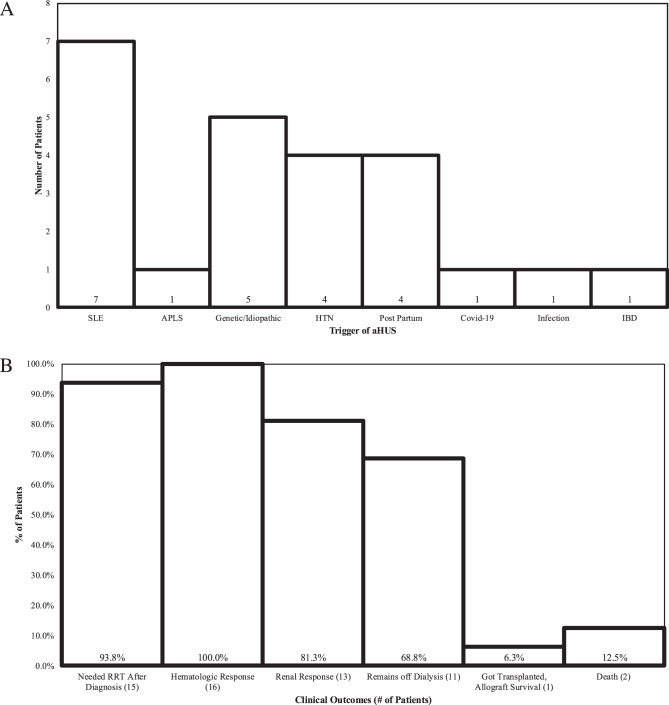
CM-TMA Triggers [n = 24]. (**a**) APLS, antiphospholipid antibody syndrome; COVID 19, novel coronavirus 2019; HTN; hypertension; IBD, inflammatory bowel disease (Crohn’s and ulcerative Colitis); SLE, systemic lupus erythematosus. (**b**) %, percent; RRT, renal replacement therapy

Of the treated aHUS subgroup, 16/16 patients (100%) demonstrated a hematological response. 13/16 (81.3%) patients showed renal response while 11/16 (68.8%) patients were able to come off dialysis and the same number remain off dialysis for greater than 12 months at this time. Of our 16 treated patients, 1 patient (6.25%) required rescue therapy with Factor B Inhibitor (iptacopan) obtained by managed access approved by USFDA after trial with eculizumab and ravulizumab. One patient (6.25%) discontinued treatment post deceased donor renal transplantation (DDRT), had recurrence, and complete remission of post-transplant aHUS recurrence when treated with C5 blockade. Of the 16 patients that were treated 2 patients expired (12.5%).

**Table 3:** CM-TMA subgroup triggers and outcomes

CM-TMA	Total (*n* = 24)
Trigger	
SLE	7 (29.2%)
Genetic/Idiopathic	5 (20.8%)
HTN	4 (16.7%)
Post-Partum	3 (12.5%)
Covid-19	2 (8.33%)
APLS	1 (4.17%)
Infection	1 (4.17%)
IBD	1 (4.17%)
Treated aHUS	**Total (n = 16)**
Outcomes	
Hematologic Recovery of Treated aHUS	16 (100%)
Needed RRT after Diagnosis	15 (93.8%)
Renal Remission with Treatment	13 (81.3%)
Remains off Dialysis	11 (68.8%)
Rescue therapy needed ^1^	1 (6.25%)
aHUS recurrence post-transplant^2^	1 (6.25%)
Had Transplant, Allograft Survival	1 (6.25%)
Death	2 (12.5%)
Non-Treated aHUS	**Total (n = 8)**
Rationale for non-treatment of aHUS	
ESRD (Renal Function Too Low)	4 (50.0%)
Peri-Transplant C5 Blockade Planned	5 (62.5%)
Sepsis/Infectious Etiology	1 (12.5%)
Plan to Start Therapy Shortly	2 (37.5%)

This table documents the subgroup of patients diagnosed with complement mediated TMA and their triggers. The table also documents the responses/outcomes of the patients treated with complement blockade and those not treated with complement blockade

aHUS = atypical Hemolytic Uremic Syndrome; APLS = Antiphospholipid; ESRD = End Stage Renal Disease; HTN = Hypertension; IBD = Inflammatory Bowel Disease; SLE = Systemic lupus erythematosus; RRT, renal replacement therapy; TMA = Thrombotic microangiopathies. Footnote 1: 1 patient with incomplete response with eculizumab, then complete remission on ravulizumab, then unexpected treatment failure with ravulizumab. Pt experienced renal and hematologic remission with factor b blockade obtained by expanded access from USFDA. Footnote 2: patient discontinued treatment post DDRT, recurrence, and complete remission of post-transplant aHUS recurrence with C5 blockade

^1^ Patient with incomplete response with eculizumab, then complete remission on ravulizumab, then unexpected treatment failure with ravulizumab. Pt experienced renal and hematologic remission with factor b blockade obtained by expanded access from USFDA. Footnote 2: patient discontinued treatment post DDRT, recurrence, and complete remission of post-transplant aHUS recurrence with C5 blockade

^2^ Patient discontinued treatment post DDRT, recurrence, and complete remission of post-transplant aHUS recurrence with C5 blockade

Of the patients who were diagnosed with aHUS CM-TMA, 16 had presentations that were active and were treated with complement inhibition, while 8 patients had presentations that were ESRD, had no active hematologic issues, had contraindications to complement blockade therapy, or who were referred for planning peri-transplant anti-complement therapy. In our subgroup of untreated aHUS patients, 8 patients (50% of untreated aHUS patients) were not treated due to already having ESRD. We have 1 patient (12.5% of untreated aHUS patients) who declined anti-complement therapy but may reconsider peri-transplant.

When there was a plan for transplant, peri-transplant blockade will be planned for these patients. [3, 17] Our protocol indicated that if a diagnosis of aHUS was suspected then, and if there was evidence of active clotting, then therapy could be offered [3, 17]. UCI plans to offer 5 patients of the 8 untreated aHUS patients peri-transplant complement blockade. 1 patient (12.5% of untreated aHUS patients) could not be treated due to ongoing sepsis, and active sepsis at our center is considered a contraindication for therapy given US FDA label. 2 patients (37.5%) are undergoing workup and to be started on anti-complement blockade shortly after vaccination and approval.

**Fig. 4:**
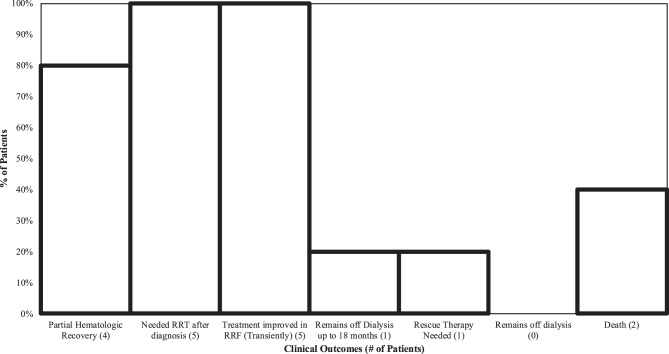
Treated HSCT-TMA outcomes [n = 5]. HSCT TMA, hematopoietic stem cell transplant TMA; RRT, renal replacement therapy; TMA, thrombotic microangiopathy

The HSCT TMA subgroup consists of 8 patients, 5 treated and 3 who were not able to be treated. All those treated were treated with the higher dose algorithm from Jodele et.al. and vaccinated as per protocol with antibiotic coverage [18–20]. Our cohort had 2 patients who could not be treated: 1 HSCT patient (12.5%) could not be treated due to sepsis, and 1 is awaiting treatment initiation (12.5%) due to scheduling and availability for the administration of therapy (Fig. 4) and (Table 4).

**Table 4:** HSCT TMA subgroup outcomes

HSCT-TMA treated	Total (*n* = 5)
Outcomes	
Partial hematologic response at any time	4 (80.0%)
Needed RRT after diagnosis^1^	5 (100%)
Treatment improved in RRF (transiently)^2^	5 (100%)
Off Dialysis up to 18 months	1 (20.0%)
Rescue therapy needed	1 (20.0%)
Remains off dialysis	0 (0.0%)
Death	2 (40.0%)
Non-Treated HSCT-TMA	**Total (n = 3)**
Rationale for non-treatment of HSCT-TMA Patients	
Total HSCT patients	8 (100%)
Awaiting Treatment	2 (25%) [66.7% untreated patients]
Active Sepsis/Infection	1 (12.5%) [33.3% untreated patients]

This table documents the outcomes of all patients with HSCT-TMA treated with complement blockade and not treated and their outcomes, HSCT TMA = hematopoietic stem cell transplant

^1^ All three patients had transient renal recovery in function including some instances transiently coming off dialysis or decreasing the frequency of dialysis

^2^ One patient renal function improvement a little with C5 inhibition (eculizumab), but improved more completely after switching to Narsoplimab – MASP-2 inhibitor

Of the 5 treated, 4 (80.0%) had a hematologic response at any time, and 100% had some renal improvement; however, no patients had a clinically meaningful prolonged response to therapy and no patients attained freedom from dialysis. Two patients (40.0%) died despite renal recovery from complications of HSCT-TMA, no patients currently remain off dialysis, 1 patient (20%) required rescue therapy with Narsoplimab a MASP-2 Inhibitor.

**Fig. 5:**
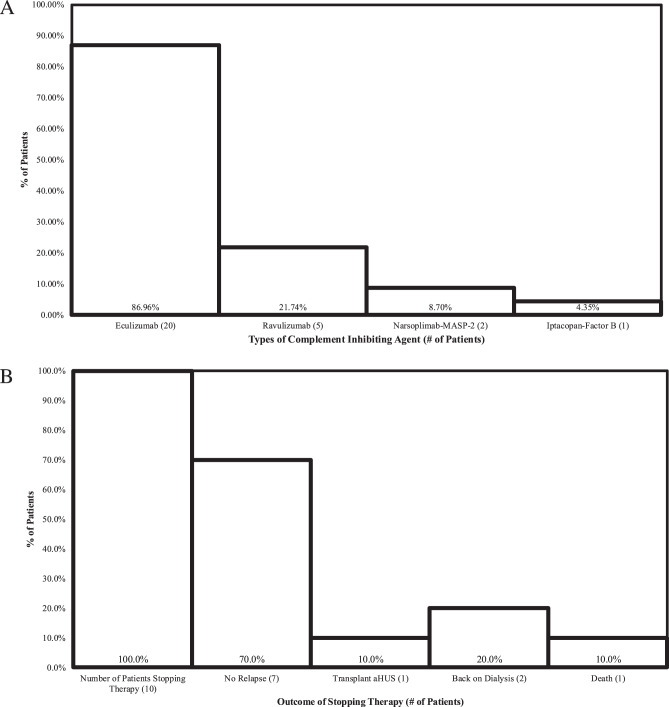
Type of complement blockade used [n = 23]. (**a**) C5, factor. Stopping complement therapies and outcomes. (**b**) aHUS, atypical hemolytic uremic syndrome

Overall, 16 aHUS patients, 5 post HSCT TMA patients, 1 drug induced TMA patient, and 1 renal limited TMA patient were treated with complement blockade in our cohort of 46 patients diagnosed with TMA. The complement inhibitor agents used in our aggregate treated cohort of 23 patients varied. In some cases, various agents were utilized due to ‘cross over’ therapy for convenience of dosing interval. In 1 of our cases, Iptacopan (a factor B inhibitor) was approved for use by USFDA due to the lack of efficiency of C5 blockade in this patient. Twenty patients were treated with eculizumab, 5 were treated with ravulizumab from this cohort of 23 treated patients, 2 were treated with Narsoplimab, and 1 treated with iptacopan. It is important to note that some patients received more than one drug in series if treatment was not optimally successful (See Fig. 5).

Ten of the patients who achieved remission in our aHUS cohort elected to stop complement therapy after a minimum of 1 year of therapy. Of those patients, 1/10 (10%) was a aHUS patient who had received a transplant and did not want to be on intravenous C5 blockade after transplant. Despite recommendations to continue, the patient insisted but was very closely monitored. Shortly after stopping, the patient recurred with AKI and a drop of platelets from 200,000 to 80,000. Both manifestations stopped immediately after restarting C5 blockade, and the patient is now stably maintained on ongoing therapy. Another patient recurred had a post-transplant aHUS recurrence, and is now back on dialysis. Yet another patient who relapsed not only required renal replacement therapy but expired thereafter. Thus, 7 patients from the group that stopped therapy successfully terminated theray, as such, a 30% relapse rate was calculated in our cohort with treatment cessation.

Table 5 looks at the results of the time to diagnosis, time from diagnosis to therapy, and total time from diagnosis to therapy for aHUS, HSCT-TMA, and other/renal TMA (encapsulating the 1 renal limited and 1 drug induced renal limited TMA that were treated). For the overall treated group, the average time to diagnosis was 13.61 days [median 7 days], average time from diagnosis to therapy was 3.7 days [median 1 day], average overall time from presentation to therapy was 17.3 days [median 8 days]. For the treated aHUS group the average time to diagnosis was 8.88 days [median 6.5 days], average time from diagnosis to therapy was 3.63 days [median 1 day], and average overall time from presentation to therapy was 12.5 days [median 7.5 days]. For the treated HSCT-TMA group, average time to diagnosis was 10.4 days [median 4 days], average time from diagnosis to therapy was 2.6 days [median 2 days], and average overall time from presentation to therapy was 13 days [median 8 days]. For the treated Other/Renal limited TMA group, the average time to diagnoses were longer (requiring the decision to biopsy) at 59.5 days [median 59.5 days]. The average time from diagnosis to therapy was 7 days [median 7 days], and the average overall time from presentation to therapy was 66.5 days [median 66.5 days]. For the sake of a concise presentation, median, and minimum-maximum range data for each time point is elaborated in Table 5.

**Table 5:** Time from diagnosis to therapy

Treated TMA type (HSCT/aHUS/RL)	Time to Diagnosis (Days)	Time to Treatment (Days)	Diagnosis to Therapy Time (Days)
aHUS	10	14	4
aHUS	24	25	1
aHUS	24	37	13
aHUS	1	1	0
aHUS	14	30	16
aHUS	27	28	1
aHUS	1	3	2
aHUS	1	1	0
aHUS	7	8	1
aHUS	6	7	1
aHUS	1	2	1
O/RL TMA	14	28	14
HSCT TMA	3	8	5
aHUS	3	4	1
aHUS	1	1	0
aHUS	10	17	7
aHUS	5	5	0
aHUS	7	17	10
HSCT TMA	33	37	4
HSCT TMA	4	6	2
HSCT TMA	8	8	0
HSCT TMA	4	6	2
O/RL TMA	105	105	0
Average time (Days)	13.61	17.30	3.70
Average time, aHUS (Days)	8.875	12.5	3.625
Average time, HSCT-TMA (Days)	10.4	13	2.6
Average time, O/RL-TMA (Days)	59.5	66.5	7
Median time (Days)	7	8	1
Median time aHUS (Days)	6.5	7.5	1
Median time HSCT-TMA (Days)	4	8	2
Median time O/RL-TMA (Days)	59.5	66.5	7
Minimum time (Days)	1	1	0
Maximum time (Days)	27	37	16
Minimum time aHUS (Days)	1	1	0
Maximum time aHUS (Days)	27	37	16
Minimum time HSCT-TMA (Days)	3	6	0
Maximum time HSCT-TMA (Days)	33	37	5
Minimum time O/RL-TMA (Days)	14	28	0
Maximum time O/RL-TMA (Days)	105	105	14

aHUS, atypical hemolytic uremic syndrome; HSCT-TMA, hematopoietic stem cell transplant (associated) thrombotic microangiopathy; O/RL-TMA, other/renal limited thrombotic microangiopathy; TMA, thrombotic microangiopathy

## Discussion

Here we present a large cohort of descriptive data documenting 101 patients, 46 with confirmed TMA. Of those with confirmed TMA, 24 with aHUS, 8 with HSCT TMA, and 14 with other types of TMA were identified. Of those, 23 patients were treated with C5 and other complement blocking agents, 16 with aHUS, 5 with HSCT TMA, and 2 with other etiologies of TMAs treated.

We also report the outcomes associated with those treated within the CM-TMA groups including morbidity, mortality, and response rates. The difficulty in treating HSCT TMA was already recognized by other groups and is published elsewhere [18–23]. We concur that due to the severe complement derangement in HSCT TMA have adjusted our protocol to dose the drug more heavily. We report the use of Narsoplimab in patients with HSCT-TMA and iptacopan in patients with aHUS after trial with eculizumab and ravulizumab. Narsoplimab was obtained under an expanded access program available through the Omeros Corporation, and iptacopan was obtained via managed access approved by USFDA through the Novartis corporation. To our knowledge, the use of iptacopan and Narsoplimab in our cohort records the first publication of experience with these agents outside of various Phase II and Phase III Trials managed access program at our center.

Our database hopes to collect large enough numbers of each type of TMA, to look at the similarities and differences of these patients’ demographics, genetics, lab values, response to therapy, and outcomes. Within the CM-TMA group perhaps differences will be found amongst different aHUS triggers. TTP, STEC-HUS, C3 GN and other unusual TMA cases were also noted. It is our ambition to build a large enough database to compare groups of patients in the various subgroups mentioned. This mirrors efforts by other TMA teams across the nation [5, 6, 24–28].

The hope is to demonstrate UCI utilizing the TMA team concept to innovate as far as diagnostic protocols, AI algorithms, databases, patient outcome registries, lab values, genetics, tissue banks, biomarker studies, and clinical trials. This manuscript is not without its limitations. As this is a descriptive manuscript, the first limitation is that this work does not establish any causal inferences or explanations; however, aims to present valuable observations and characteristics of the TMA disease process and the operations of a multidisciplinary team. Another limitation in our study is that our study does not include solid organ transplant associated TMA. Another limitation includes the absence of other data that is necessary but hard to collect including data on discontinuation of drug and outcomes. Although there are many emerging complements testing and genetic testing assays which have improved in quality over the past year, the current lack of adequate and specific tests remains a limitation for definitive diagnosis of complement and genetic involvement. Our study, although limited by our small sample size, notes a high rate of relapse in our TMA patients. These findings are consistent with previous literature, which has documented variations in relapses rates dependent on the specific characteristics of the patient cohort under investigation [29–32]. The aim of the UCI TMA team is to investigate aHUS, and as new treatments and diagnostics become available to leverage this data to help better classify TMA patients to allow better innovation, research, and diagnosis. National Institute of Health (NIH) funding, as well as industry is being considered to grow the UCI TMA team, efforts are being made to also partner with industry and other major medical centers to form a US national registry. Our aim is to gather further long-term data to perform greater robust statistical analysis with distinctive statistical power.

Improving the procedures of our own TMA team and future TMA teams is a top priority. TMAs are very difficult diagnoses to make when they present typically. It is known that aHUS and other TMAs do not always present typically. There are cases where thrombocytopenia is not present or not prominent. Other cases where alternative causes can explain the hemolysis but misses the TMA picture. We estimate at least 25% of cases may present atypically, thus there is a large enough portion of patients that dictate an ongoing refining of the TMA team processes, algorithms, and electronic medical record order sets.

Capturing these atypical cases can be achieved by three major ways, GAI (General Artificial Intelligence) algorithms which we are working with some sponsors and our EMR to implement. The other way is to increase education amongst our staff- a task we are tirelessly undertaking. Finally, the search for a biomarker would be greatly helpful, and that is why we are partnering with diagnostics labs on the use of C5b-9 and a modified HAM (Hemolytic Anemia Modified) 2.0 assay.

## Conclusion

This observational study lists the components of UCI TMA multidisciplinary team, its algorithms for TMA diagnosis, and 4 years of data of this rare disease process. Our TMA multidisciplinary team dataset has potential to grow into a regional resource and eventually we hope it will be a nidus for a national TMA database [1]. The possibility of tissue banking and patient outcomes tracking makes clinical trials, diagnostics research, basic science research expansion feasible. We hope this approach will continue to allow UCI to develop into a regionally and nationally recognized TMA center of excellence [1, 3, 4, 7–12]. With acquisition of further data, we aim to conduct more robust statistical analysis regarding outcomes of various classes of complement drugs. This database will allow translational medicine for TMA patients similar to the scale found in Europe, but here in the United States.

In the meanwhile, we the authors, would encourage every tertiary care university center to form an ‘as needed’ TMA team, keep meticulous records of cases under institutional review board oversight, and eventually to convert it to a standing TMA board-managed similarly to a multidisciplinary tumor board model seen in oncology.

## Electronic supplementary material

Below is the link to the electronic supplementary material.

Supplementary Material 1

Supplementary Material 2

## Data Availability

All data generated or analyzed during this study are included in this published article and its supplementary information files.
