# Supplementary material for: Thrombotic microangiopathy multidisciplinary assessment team: demographics, final diagnosis, treatment, and outcomes
Source: BMC Nephrol. 2025 Sep 26;26:537. doi: 10.1186/s12882-025-04446-z (PMC12465760; doi:10.1186/s12882-025-04446-z)

Supplemental Figure 1a Gross Demographic Histogram of Diagnosis [n = 101]

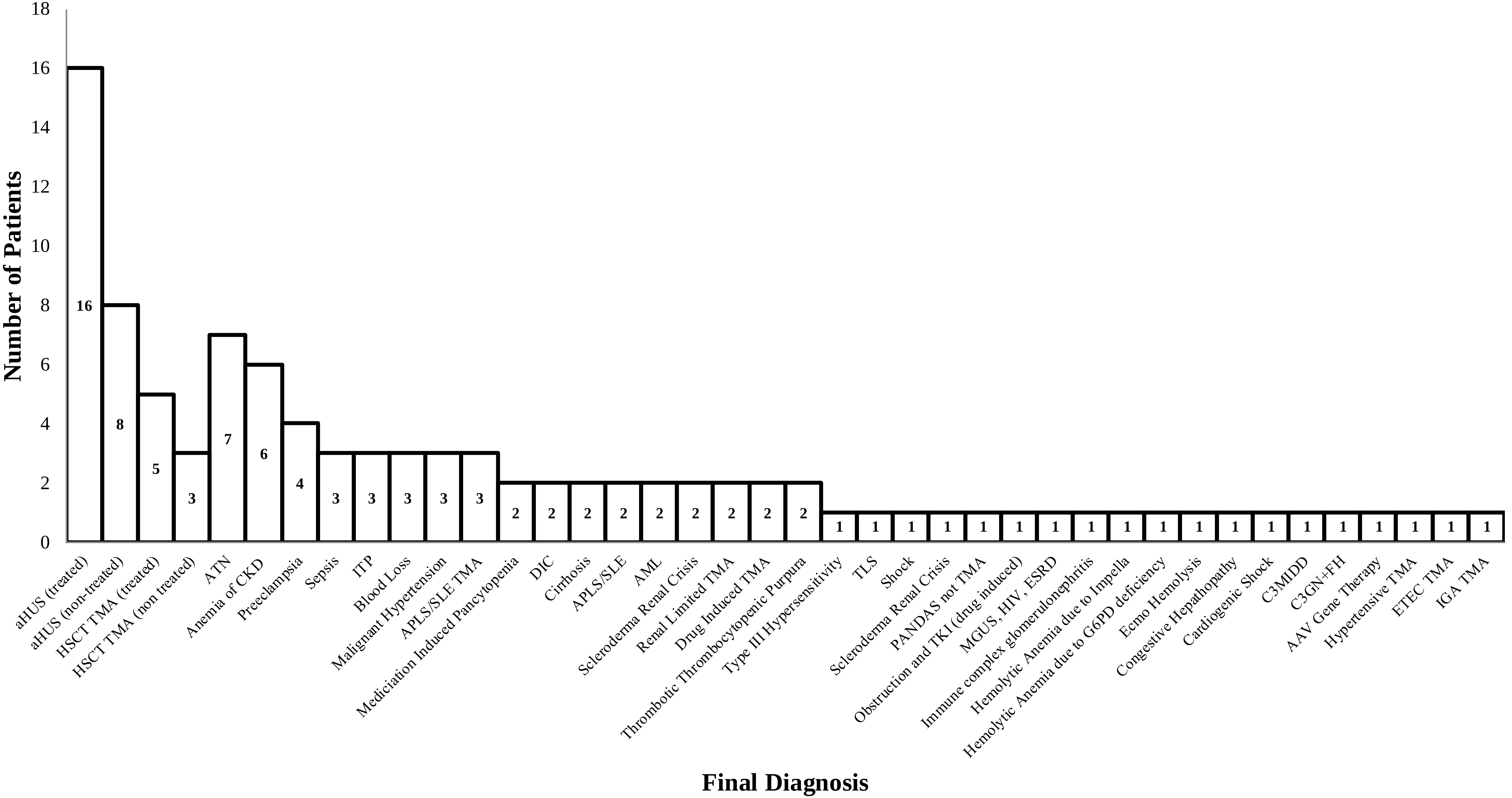

Supplemental Figure 1b Age/Gender Demographics [n = 101]

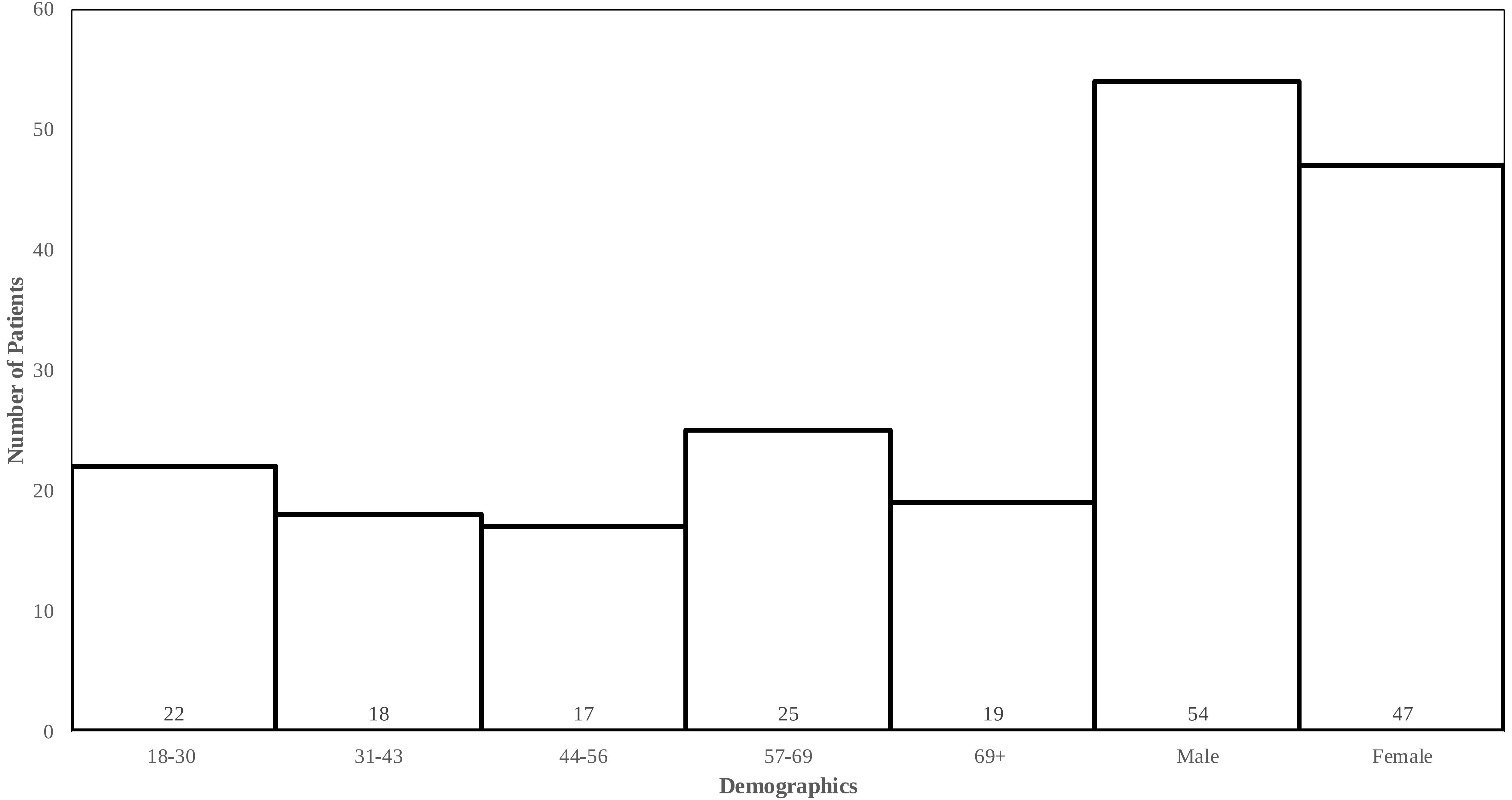

Supplemental Figure 2 Non-Treated aHUS – Rationale [n = 8]

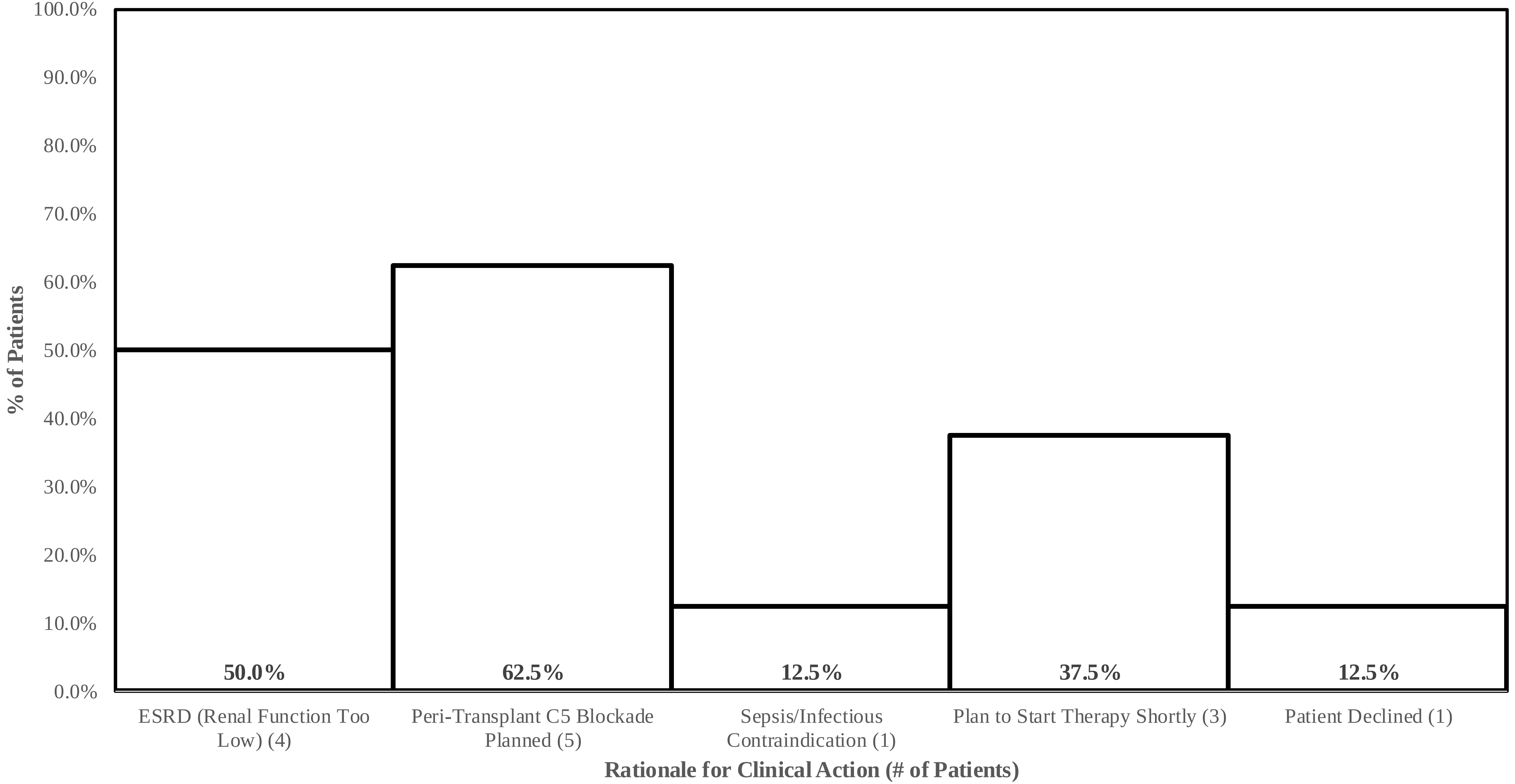

Supplemental Figure 3 Non-Treated HSCT-TMA - Rationale [n = 3]

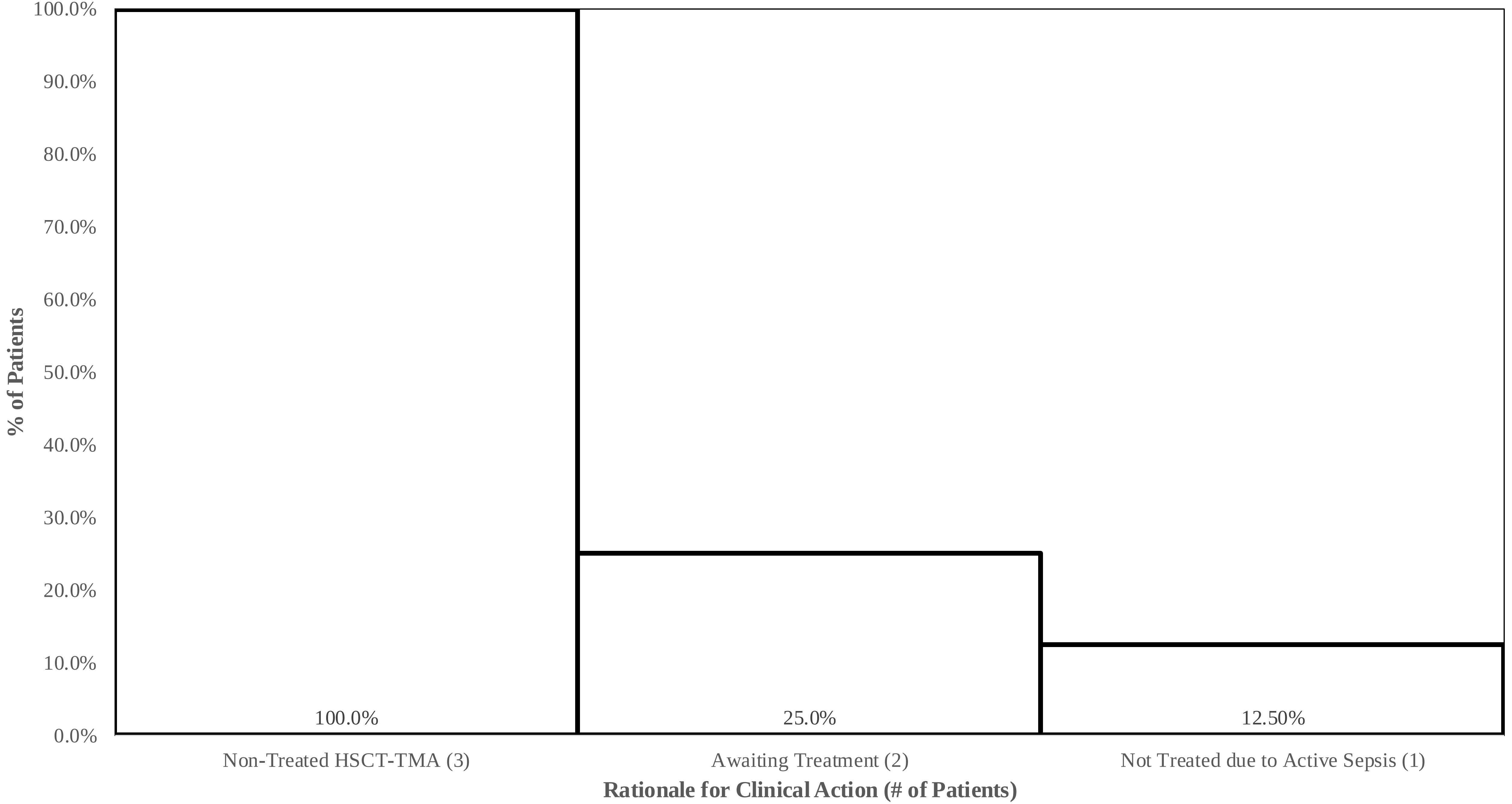

Supplemental Figure 4 Indications for Complement Blockade [n = 23]

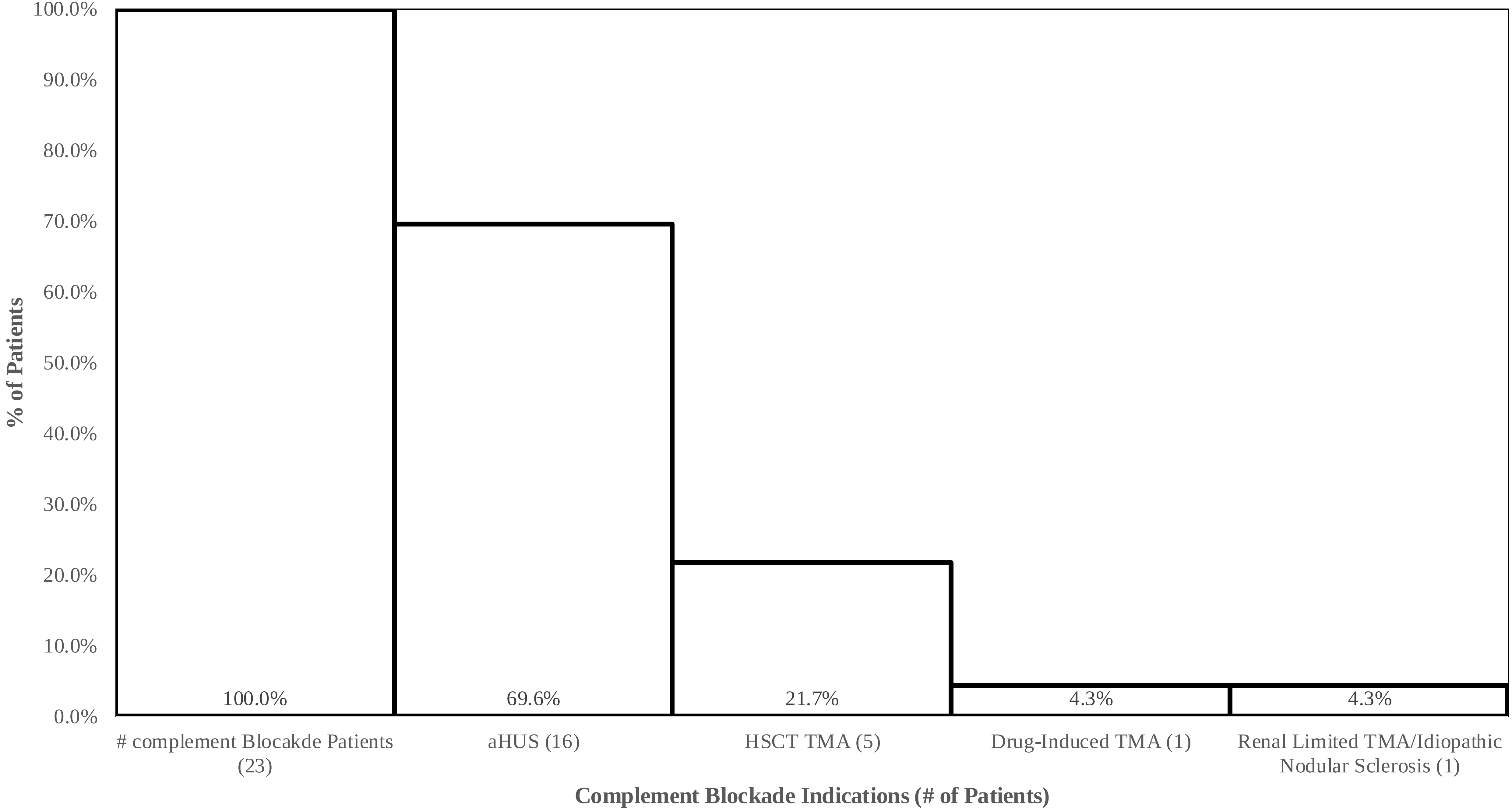

Supplement: Supplementary file 1 — Supplementary Material 1 [file 12882_2025_4446_MOESM1_ESM.pdf]
