# Supplementary material for: Thrombotic microangiopathy multidisciplinary assessment team: demographics, final diagnosis, treatment, and outcomes
Source: BMC Nephrol. 2025 Sep 26;26:537. doi: 10.1186/s12882-025-04446-z (PMC12465760; doi:10.1186/s12882-025-04446-z)
Supplement: Supplementary file 2 — Supplementary Material 2 [file 12882_2025_4446_MOESM2_ESM.docx]

**Supplemental Figure 1 legend:**

**s2a.** aHUS, atypical hemolytic uremic syndrome; HSCT, hematopoietic stem cell transplant; TMA, thrombotic microangiopathy; ATN, acute tubular necrosis; CKD, chronic kidney disease; ITP, immune thrombocytopenia purpura; APLS, antiphospholipid syndrome; SLE, systemic lupus erythematosus; DIC, disseminated intravascular coagulation; AML, Acute myelogenous leukemia; TLS, tumor lysis syndrome; PANDAS, Pediatric Autoimmune Neuropsychiatric Disorders Associated with Streptococcal Infections; TKI, tyrosine kinase inhibitor; MGUS, monoclonal gammopathy of undetermined significance; HIV, human immunodeficiency virus; ESRD, end stage renal disease; G6PD, glucose 6 Phosphate Dehydrogenase Deficiency (hemolysis); ECMO, extracorporeal membrane oxygenation; C3MIDD, C3 monoclonal immunoglobulin deposition disease; C3GN, C3 glomerulonephritis; FH, Factor H; AAV, adeno associated virus; ETEC, Enterotoxigenic Escherichia coli; IGA, immunoglobulin A

**s2b** (no legend needed).

**Supplemental Figure 2 legend:**

aHUS, atypical hemolytic uremic syndrome; ESRD, End-stage renal disease

**Supplemental Figure 3 legend:**

HSCT TMA, hematopoietic stem cell transplant thrombotic microangiopathy

**Supplemental Figure 4**

aHUS, atypical hemolytic uremic syndrome; HSCT, hematopoietic stem cell transplant; TMA, thrombotic microangiopathy
